# Pharmacological interventions for challenging behaviour in children with intellectual disabilities: a systematic review and meta-analysis

**DOI:** 10.1186/s12888-015-0688-2

**Published:** 2015-11-26

**Authors:** Cheryl McQuire, Angela Hassiotis, Bronwyn Harrison, Stephen Pilling

**Affiliations:** 1 National Collaborating Centre for Mental Health, Royal College of Psychiatrists, 21 Prescot Street, London, E1 8BB UK; 2 Division of Psychiatry, University College London, Charles Bell House, 1st and 2nd Floor, 67-73 Riding House Street, London, W1W 7EJ UK; 3 Centre for Outcomes Research and Effectiveness, University College London, 1-19 Torrington Place, London, WC1E 7HB UK

**Keywords:** Intellectual disabilities, Challenging behaviour, Pharmacological treatment, Antipsychotics, Systematic review, Meta-analysis, Randomised controlled trials

## Abstract

**Background:**

Psychotropic medications are frequently used to treat challenging behaviour in children with intellectual disabilities, despite a lack of evidence for their efficacy. This systematic review and meta-analysis aimed to determine the safety and efficacy of pharmacological interventions for challenging behaviour among children with intellectual disabilities.

**Methods:**

Electronic databases were searched and supplemented with a hand search of reference lists and trial registries. Randomised controlled trials of pharmacological interventions for challenging behaviour among children with intellectual disabilities were included. Data were analysed using meta-analysis or described narratively if meta-analysis was not possible. For quality assessment, the Cochrane Risk of Bias tool and the Grading of Recommendations Assessment, Development and Evaluation (GRADE) approach were used.

**Results:**

Fourteen studies including 912 participants met inclusion criteria. Antipsychotic medication reduced challenging behaviour among children with intellectual disabilities in the short-term (SMD = −1.09, *p* < 0.001 for risperidone; SMD = −0.64, *p* <0.001 for aripiprazole). However, there were significant side-effects including elevated prolactin levels (SMD = 3.22, *p* < 0.001) and weight gain (SMD = 0.82, *p* < 0.001). Evidence was inconclusive regarding the effectiveness of anticonvulsants and antioxidants for reducing challenging behaviour. The quality of all evidence was low and there were no long term follow up studies.

**Conclusions:**

Antipsychotic medications appear to be effective for reducing challenging behaviour in the short-term among children with intellectual disabilities, but they carry a risk of significant side effects. Findings from this review must be interpreted with caution as studies were typically of low quality and most outcomes were based on a small number of studies. Further long-term, high-quality research is needed to determine the effectiveness and safety of psychotropic medication for reducing challenging behaviour.

**Electronic supplementary material:**

The online version of this article (doi:10.1186/s12888-015-0688-2) contains supplementary material, which is available to authorized users.

## Background

Intellectual disability or intellectual developmental disorder (IDD) is characterised by an impairment of mental ability and adaptive functioning that originates in childhood [1]. One per cent of the population are thought to have IDD [2]. Children, young people and adults with IDD are vulnerable to developing mental ill health and face health inequalities [3, 4]. A common problem in this population is challenging behaviour, which presents as episodic aggression towards others and the environment, self-injury, and a host of other behaviours that may be seen as socially unacceptable and prevent the individual from fully participating in day to day life [5]. The prevalence of such problems is lower in the community than in hospital or congregate settings but rates vary according to features of study design, such as how challenging behaviour is measured [6]. Challenging behaviour is one of the factors leading to the exclusion of individuals from their local communities, precipitation of abusive practices and poorer quality of life for the individual and the family carers [7–9]. Staff caring for individuals with IDD and challenging behaviour may report burnout and low job satisfaction [10, 11].

A variety of psychosocial interventions have been used in the treatment and management of challenging behaviour but often medication may be added, or may be used as the sole treatment [12]. It is thought that psychotropic medication, particularly antipsychotic drugs, are prescribed all too often for these individuals and may be administered long-term [13–15].The justification for their use in the absence of an ICD or DSM psychiatric disorder is commonly cited as an improvement in mood dysregulation. However, such drugs have significant side effects including the metabolic syndrome, movement disorders, such as drug induced Parkinsonism and tardive dyskinesia, and osteoporosis [16–19]. The impact of psychotropic medication on challenging behaviour is less well established [20].

A multinational study using the IMS Prescribing Insights database investigated prescriptions for children and adults with Autism Spectrum Disorders (ASD). The authors found that prescriptions for children far outnumbered those for adults with the most commonly prescribed medication being risperidone; although the class of drug varied across countries [21, 22]. A further investigation of the use of psychotropic medication for children and young persons aged 0–24 years with ASD in primary care in the UK indicated that approximately 12.6 % had a comorbid intellectual disability and that psychostimulants and hypnotics were prescribed most often [23]. The findings further showed that there was an increase in prescriptions issued over a period of 16 years.

Given the risks and substantial prescribing of psychotropic medications, for a population who are known to face health inequalities and who are unable to identify symptoms of disease or seek help early, it is paramount that evidence is gathered to inform routine care and improve standards [24].

### Aims of the study

This review aims to summarise the available empirical evidence regarding the efficacy and safety of pharmacological interventions for children with intellectual disabilities and challenging behaviour.

There are currently no systematic reviews that comprehensively evaluate the broad range of pharmacological interventions that have been used with this population. Therefore, it is hoped that the findings from the review and meta-analysis will assist in guiding clinicians and researchers to optimise prescription patterns for a vulnerable population group.

## Methods

This review and meta-analysis followed methodological and reporting guidelines from the National Institute for Health and Care Excellence (NICE) [25], Cochrane Handbook for Systematic Reviews of Interventions [26] and Preferred Reporting Items for Systematic Reviews and Meta-Analyses (PRISMA; http://www.prisma-statement.org/). A completed PRISMA checklist is available (see Additional file 1).

Ethical approval was not required as the study was a systematic review. The full review protocol is available from https://www.nice.org.uk/guidance/ng11.

### Eligibility Criteria

#### Types of trials

Studies were included if they were randomised controlled trials (RCTs) or cluster RCTs with at least 10 participants per arm.

#### Types of participants

Eligible participants were children and young people up to the age of 18 years with intellectual disabilities and challenging behaviour. Intellectual disabilities were defined by the following criteria: an intelligence test score of less than 70, significant impairment of social or adaptive functioning, and onset in childhood. This corresponds to ’mental retardation’ as described in the major taxonomies DSM-5 and ICD-10 [1, 27]. Studies involving children with autism but which did not explicitly report co-occurring intellectual disability were included unless it was clear that participants had high-functioning autism. This decision was based on evidence suggesting that 50–70 % of individuals with autism spectrum disorders also have intellectual disabilities [28, 29]. Challenging behaviour was defined as “culturally abnormal behaviour(s) of such an intensity, frequency or duration that the physical safety of the person or others is likely to be placed in serious jeopardy, or behaviour which is likely to seriously limit use of, or result in the person being denied access to, ordinary community facilities (p.4)” [5].

#### Types of interventions

Eligible interventions were pharmacological interventions aimed at reducing or managing challenging behaviour, compared with treatment as usual, placebo or an alternative active intervention.

### Outcomes of interest

#### Critical outcomes

Critical efficacy outcomes were targeted challenging behaviour, adaptive functioning, quality of life, and service user and carer satisfaction. Critical safety outcomes were adverse events including sedation and somnolence, weight and prolactin outcomes, seizures, and study discontinuation.

#### Secondary outcomes

Secondary outcomes were mental and psychological health outcomes, effects on carer stress and resilience, adverse effects on other people with intellectual disabilities, rates of seclusion, rates of manual restraint, use of psychoactive medication, premature death, rates of placement breakdown and use of inpatient placements.

### Search strategy

Electronic databases including Embase, Medline, PreMedline, PsycINFO, Cochrane Central Register of Controlled Trials (CENTRAL), Cochrane Database of Systematic Reviews (CDSR), Cochrane DatabaseofAbstractsofReviewsofEffects(DARE), Sociological Abstracts, Social Services Abstracts, Education Resources Information Centre (ERIC), British Education Index (BEI), International Bibliography of the Social Sciences, (IBSS) and the Social Sciences Citation Index (SSCI) were searched from their inception until October 2014. We searched for unpublished evidence using the NICE call for evidence process [25], via a search of clinicaltrials.gov and through contact with subject experts who were identified by members of the guideline development group and prior literature searches. Additional searches included a hand search of study reference lists. The search for pharmacological evidence formed part of a broader search for clinical trials on intellectual disabilities and challenging behaviour. Full details of the search strategy for Medline can be found in Additional file 2. This search string was translated for use in all other databases listed.

Studies were screened for eligibility by two authors (CM and BH) and on occasions where consensus could not be reached a third author (SP) was consulted to determine inclusion. Studies investigating drugs that were no longer licensed, or involving drugs for which there was no sound theoretical basis for their use in the treatment of challenging behaviour, such as the cough suppressant Dextromethorphan, were excluded based on consultation with a chief pharmacist. Studies were excluded if participants had co-existing conditions, including major mental disorders such as schizophrenia. In cases where some, but not all, of a study’s participants were eligible for the review - such as a mixed population of individuals with and without intellectual disability - study authors were asked to provide disaggregated data for the subset of participants who met inclusion criteria. Non-English language publications were excluded unless an English version could be located. Conference abstracts and dissertations were excluded.

Findings from studies exploring sleep problems, drug discontinuation and those investigating biomedical interventions, such as dietary supplements, form part of a separate review and are available in the full NICE guideline for challenging behaviour and learning disabilities [30].

### Data management

Outcome data were extracted into an electronic database by one author (CM) and assessed for accuracy by a second author (BH). The database captured information about participant demographics, intervention characteristics, study methodology, funding sources, and missing data. Authors were contacted for further information if data or study characteristics were not available.

### Statistical analysis

Data were analysed with random effects meta-analysis using the Review Manager software (RevMan, Cochrane, Copenhagen) [31]. The standardised mean difference (SMD; Hedges’ *g*) was used as the effect size for continuous outcomes and studies were weighted using the inverse of variance. The SMD is a summary statistic that allows outcomes from studies to be combined in meta-analysis, regardless of the original scale of measurement. This is useful in circumstances, such as the present review, where studies use different psychometric tests (for example, the Aberrant Behavior Checklist and the Nisonger Child Behavior Rating Form) to assess the outcome of interest. SMD is calculated as the difference in mean outcome between groups, divided by the standard deviation of the outcome among participants. SMDs of 0.20 were considered small, 0.50 medium and 0.80 or more large following Cohen’s criteria [32]. For dichotomous outcomes, the risk ratio (RR) was used as the measure of effect and studies were analysed with the Mantel-Haenszel method [33]. The risk ratio indicates the multiplication of the risk of an outcome in one group compared to another. As estimates of effect size can be misleading when event rates are low, outcomes with higher baseline risk (for example non-improvement rather than improvement of challenging behaviour and non-occurrence rather than occurrence of adverse events) were selected for analyses [34].

Statistical heterogeneity was assessed with the I^2^ and chi-squared (χ^2^) statistics and by visual inspection of forest plots. A *p* value of less than 0.10 or an I^2^ value of 40 % or higher was taken to indicate significant statistical heterogeneity. In the event of significant heterogeneity, the protocol pre-specified study characteristics for further investigation including the degree of learning disability in the sample and the form of challenging behaviour. The findings of subgroup investigations will only be presented if we were able to determine a plausible reason for heterogeneity. Otherwise, heterogeneity was considered unexplained. The use of random-effects models for meta-analysis reflects the assumption of unexplained heterogeneity in findings.

Intent-to-treat was the preferred method of analysis. In cases where studies reported data only for participants who completed the intervention (i.e. a completer analysis), sensitivity analyses were conducted using intent-to-treat principles, including all participants randomized into the study and using non-improvement as a worst case outcome scenario. Results of sensitivity analyses will only be reported if the effect size differs significantly from the original effect estimate.

### Risk of bias and quality assessment

We used The Cochrane Collaboration Risk of Bias Tool [26] to assess the quality of each study. Risk of bias was rated as low, high or unclear for each of the following domains: sequence generation; allocation concealment; blinding of participants, assessors, and providers; selective outcome reporting; and incomplete data. Risk of bias was assessed independently by two authors (CM and BH) and disagreements resolved following discussion with a third author (SP). Authors were contacted to request any missing information pertaining to risk of bias assessments.

The Grading of Recommendations Assessment, Development and Evaluation (GRADE) approach was used to assess the quality of evidence for each outcome [35]. Briefly, the GRADE approach considers factors including risk of bias; inconsistency; indirectness; imprecision; and publication bias, when judging the quality of evidence. When evidence is deemed to be high quality, the implication is that further research is very unlikely to change the confidence in the estimate of effect. A judgement of moderate quality suggests that further research is likely to have an important impact on the confidence in the estimate of effect and may change the estimate. Low quality evidence suggests that further research is very likely to have an important impact on the confidence in the estimate of effect and is likely to change the estimate, and a judgement of very low quality evidence indicates that the effect estimate is very uncertain.

## Results

### Results of the search

**Fig. 1:**
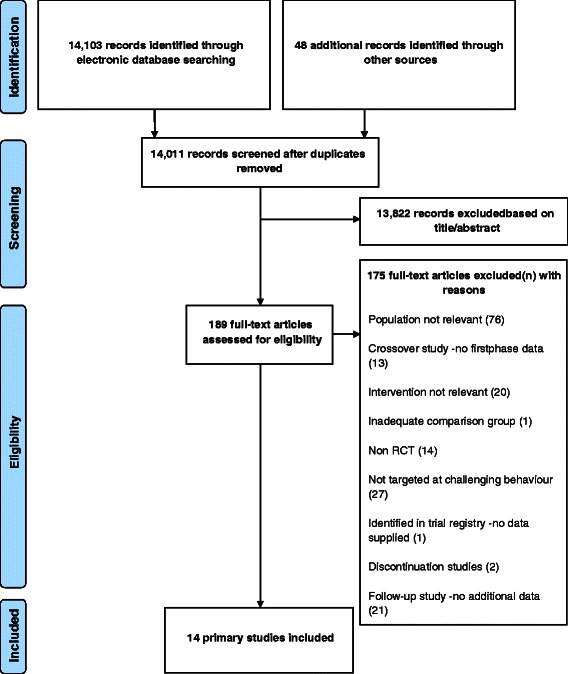
PRISMA flow chart depicting selection of studies

The search process identified 14,151 records for screening. From this, 14 primary studies were eligible for review. Figure 1 depicts a flow diagram of the selection process.

### Study characteristics

Eligible studies ranged in date from 2001–2013 and included a total of 912 participants. All but one study provided sufficient data for meta-analysis. The results of this study will be presented narratively [36]. Of the studies included in the review, nine were from the USA [37–45], three were from Iran [36, 46, 47], one was from Canada [48] and one was conducted across multiple countries [49]. Antipsychotics were the most common class of drug and were evaluated in nine studies [37, 41–46, 48, 49]. Anticonvulsants were evaluated in three studies [39, 40, 47], antioxidants in one study [38] and gamma-aminobutyric acid (GABA) analogues in one study [36]. Duration of treatment ranged from six to twelve weeks. The mean age of participants was 9 years (range 7 – 11 years) and 21 % of the sample were female. Twelve studies included participants with an autistic spectrum disorder [36, 38–48] and co-existing intellectual disability was explicitly reported in four of these studies [39, 42, 45, 48]. Two studies included children with mild to moderate intellectual disabilities [37, 49]. Twelve studies compared pharmacological treatment to placebo [36–41, 43–45, 47–49] and two conducted head-to-head trials comparing antipsychotic medication [42, 46]. All studies measured outcomes immediately following the end of treatment and none reported long-term follow-up data. No data were available for any secondary outcomes. There were no studies that met inclusion criteria with respect to pharmacological interventions for self-injurious behaviour in children.

Further characteristics of included studies are shown in Table 1.

**Table 1:** Characteristics of included studies

Study	Diagnosis	Targeted CB^a^(measure)	Intervention (dose mg/day)	Comparison (dose mg/day)	Treatment duration
Akhondzadeh 2008	ASD	Severely disruptive symptoms (ABC total)	Piracetam (800) + risperidone (3)	Placebo + risperidone (3)	10 weeks
Aman 2002	Mild to moderate ID	Conduct problems (NCBRF conduct)	Risperidone (1.2)	Placebo	6 weeks
Ghanizadeh 2013	ASD	Irritability (ABC-I)	Aripiprazole (5.5)	Risperidone (1.1)	8 weeks
Hardan 2012	ASD	Irritability (ABC-I)	N-acetylcysteine (2700)	Placebo	12 weeks
Hellings 2005	ASD + ID^b^	Irritability (ABC-I)	Valproate (20)^c^	Placebo	8 weeks
Hollander 2010	ASD^d^	Irritability (ABC-I)	Valproate (375)	Placebo	8 weeks
Kent 2013^e^	ASD	Irritability (ABC-I)	Risperidone (1.8)^f^	Placebo	6 weeks
Malone 2001	ASD + mild to severe ID^g^	Hyperactivity (CPRS-H)	Olanzapine (10)^f^	Haloperidol (2.5)	6 weeks
Marcus 2009	ASD	Irritability (ABC-I)	Aripiprazole (10)^h^	Placebo	8 weeks
Owen 2009	ASD	Irritability (ABC-I)	Aripiprazole (8.9)	Placebo	8 weeks
Rezaei 2010	ASD	Irritability (ABC-I)	Topiramate (200) + risperidone (2)^f^	Placebo + risperidone (2)^f^	8 weeks
RUPP 2002^i^	ASD + mild to severe ID	Irritability (ABC-I)	Risperidone (1.8)	Placebo	8 weeks
Shea 2004	ASD + mild to moderate ID	Conduct problems (NCBRF conduct)	Risperidone (1.5)	Placebo	8 weeks
Snyder 2002	Mild to moderate ID^j^	Conduct problems (NCBRF conduct)	Risperidone (1)	Placebo	6 weeks

Notes: ASD = autism spectrum disorder; CB = challenging behaviour; ID = intellectual disability; N = number randomised; ABC total = Aberrant Behaviour Checklist total score; ABC-I = Aberrant Behaviour Checklist: Irritability; CGI-I = Clinical Global Impressions: Improvement; CPRS-H Children’s Psychiatric Rating Scale: Hyperactivity; NCBRF = Nisonger Child Behaviour Rating Form: Conduct problems. ^a^ Primary outcome measure for meta-analysis; ^b^ 13 % of sample had borderline to average intelligence; 87 % were diagnosed with ID; ^c^ 20 mg/kg/day; ^d^15% of sample had Asperger’s syndrome; ^e^ Three armed trial: high dose risperidone and placebo arms used in meta-analysis; ^f^ Maximum dose; ^g^ 8 % of participants had normal cognitive functioning, all others had mild to severe ID; ^h^ Data from high, moderate and low dose conditions combined in meta-analyses; ^i^ Research Units on Pediatric psychopharmacology (RUPP) Autism Spectrum Disorder Network; ^j^ 2 % of participants had borderline intellectual functioning; all others had mild to moderate ID.

### Risk of bias for included studies

**Fig. 2:**
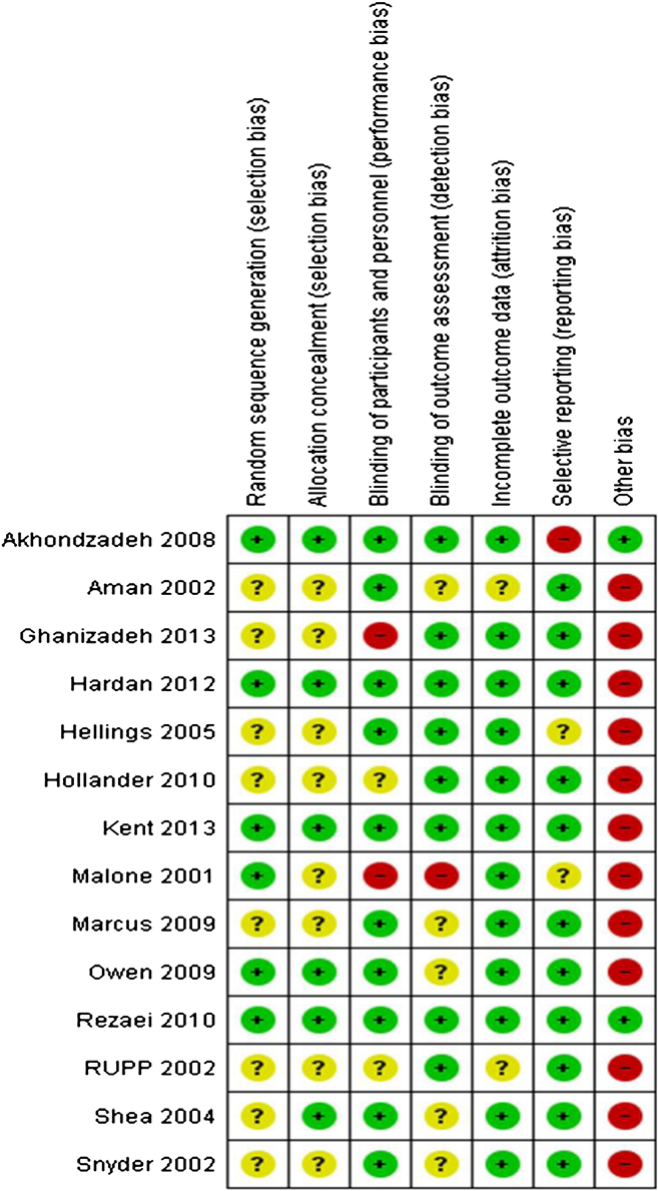
Risk of bias summary for included studies

Figure 2 provides an overview of the risk of bias assessment for included studies.

Randomisation procedures were adequate in six studies and unclear in eight studies. There was a low risk of bias for allocation concealment in six of the studies and concealment was unclear for the eight remaining studies. The majority of studies carried out adequate procedures to ensure that participants and study personnel were blind to treatment status. However, for two studies blinding was unclear and risk of bias was high for the remaining two studies, as one had an open label design and the other did not blind study personnel. In terms of blinding of outcome assessment, eight studies were deemed to be low risk of bias, five were unclear and one open label trial was considered to be at a high risk of bias. There was a low risk of bias for selective outcome reporting in 11 studies, an unclear risk for two studies and a high risk in one study. Twelve of the 14 studies were deemed to be at a high risk of bias for other reasons; most commonly because of affiliations with pharmaceutical companies, the exclusion of participants who had previously been unresponsive to medication and between group differences in concomitant medication.

Based on the GRADE approach, the quality of evidence ranged from low to very low for all outcomes. This was mainly due to small sample sizes and risk of bias within studies. Full GRADE profiles for included evidence can be found in Additional file 3.

### Efficacy and safety outcomes by drug class

#### Antipsychotics

Antipsychotics were evaluated in nine studies [37, 41–46, 48, 49].The types of drugs investigated were risperidone, aripiprazole, haloperidol and olanzapine.

#### Effectiveness outcomes

##### Challenging behaviour

Five studies with 325 participants compared risperidone to placebo [37, 41, 45, 48, 49]. Challenging behaviour was assessed using The Nisonger Child Behavior Rating Form (NCBRF) Conduct Problems subscale [50] in three studies [37, 48, 49] and the Aberrant Behavior Checklist Irritability Subscale (ABC-I) [51] and Clinical Global Impressions-Improvement (CGI-I) [52] in two studies [41, 45].

Three of these studies included children with autism who did not have intellectual disability [37, 48, 49].For these studies, we obtained disaggregated data sets from authors which included data only from children with intellectual disabilities and used these for analyses.

**Fig. 3:**
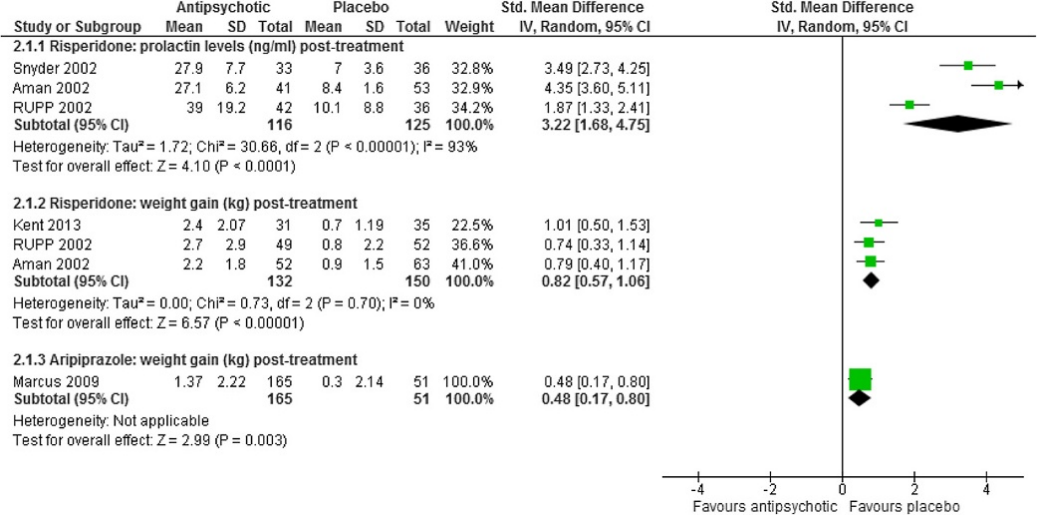
The effect of risperidone and aripiprazole on challenging behaviour in children with intellectual disabilities

Risperidone reduced challenging behaviour in comparison to placebo based on endpoint (SMD = −1.09, 95 % confidence interval [CI] -1.39 to −0.79, z = 7.07, *p* < 0.001) and change from baseline scores (SMD = −0.98, CI −1.49 to −0.47, *p* < 0.001). Results are depicted in Fig. 3. Dichotomous measures of improvement, defined as a 25 % improvement in ABC-I scores and a CGI-I rating of much improved or very much improved provided further support for the effectiveness of risperidone in reducing challenging behaviour, relative to placebo (RR = 0.42, CI 0.28 to 0.64, *p* < 0.001).

Two studies with 316 participants compared the effectiveness of aripiprazole to placebo in the treatment of challenging behaviour [43, 44]. Aripiprazole was found to be superior to placebo in terms of reducing the severity of challenging behaviour based on ABC-I scores (SMD = −0.64, CI −0.91 to −0.36, *p* < 0.001) and dichotomous improvement outcomes, classed as a 25 % improvement in ABC-I scores and a CGI-I rating of much improved or very much improved (RR = 0.65, CI 0.50 to 0.84, *p* = 0.001) (see Fig. 3).

One head-to head study compared the effectiveness of aripiprazole to risperidone [46] and found that children who received risperidone showed lower levels of challenging behaviour based on the ABC-I than those given aripiprazole, however the precision of this estimate was poor and statistical estimates of effect were inconclusive (SMD = 0.38, CI −0.14 to 0.90, *p* = 0.15).

A small open label study [42], involving 12 participants indicated that children who received olanzapine showed lower levels of challenging behaviour at the end of treatment than those who received haloperidol (SMD = −1.40, CI −2.73 to −0.08, *p* = 0.04).

##### Adaptive functioning

Three studies [37, 48, 49] provided information on the effect of risperidone on participants’ adaptive social functioning. Analysis demonstrated a large effect size (SMD = 0.86, CI 0.42 to 1.30, *p* = <0.001) suggesting that risperidone improved children’s adaptive social functioning when compared to placebo. However, there was evidence of statistical heterogeneity (χ ^2^ = 3.34, *p* = 0.19, I^2^ = 40 %), which were not readily explained by differences in study characteristics, and therefore results should be interpreted with caution.

##### Quality of life

Children who received aripiprazole showed higher quality of life scores at the end of treatment than those given placebo [43, 44]. However, confidence intervals crossed the line of no effect and, thus, the estimate of effect was inconclusive (SMD = 0.60, CI −0.17 to 1.37, *p* = 0.13). Furthermore, there was significant unexplained statistical heterogeneity for this effect (χ^2^ = 6.34, p = 0.01, I^2^ = 84 %) suggesting caution in interpretation.

##### Service user and carer satisfaction

There were no data available for the effect of any antipsychotic medication on service user and carer satisfaction.

#### Safety outcomes

##### Weight

**Fig. 4:**
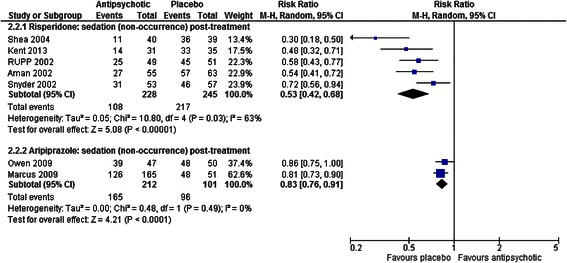
The effect of aripiprazole and risperidone on weight and prolactin levels in children with intellectual disabilities and challenging behaviour

Risperidone was associated with greater weight gain than placebo [37, 41, 45, 48, 49](SMD = 0.82, CI 0.57 to 1.06, *p* < 0.001; RR 0.91, CI 0.85 to 0.96, *p* = 0.002; see Fig. 4).

Aripiprazole was associated with greater levels of weight gain [43] (SMD = 0.48, CI 0.17 to 0.80, *p* = 0.003; see Fig. 4) and increased the risk of clinically significant weight gain when compared with placebo at the end of intervention [43, 44] (RR = 0.79, CI 0.71 to 0.88, *p* < 0.001).

Olanzapine was found to increase weight gain to a greater extent than haloperidol [42], although the precision of this estimate was poor, possibly as a result of small sample size (SMD = 1.26, CI −0.03 to 2.54, *p* = 0.06).

##### Sedation

**Fig. 5:**
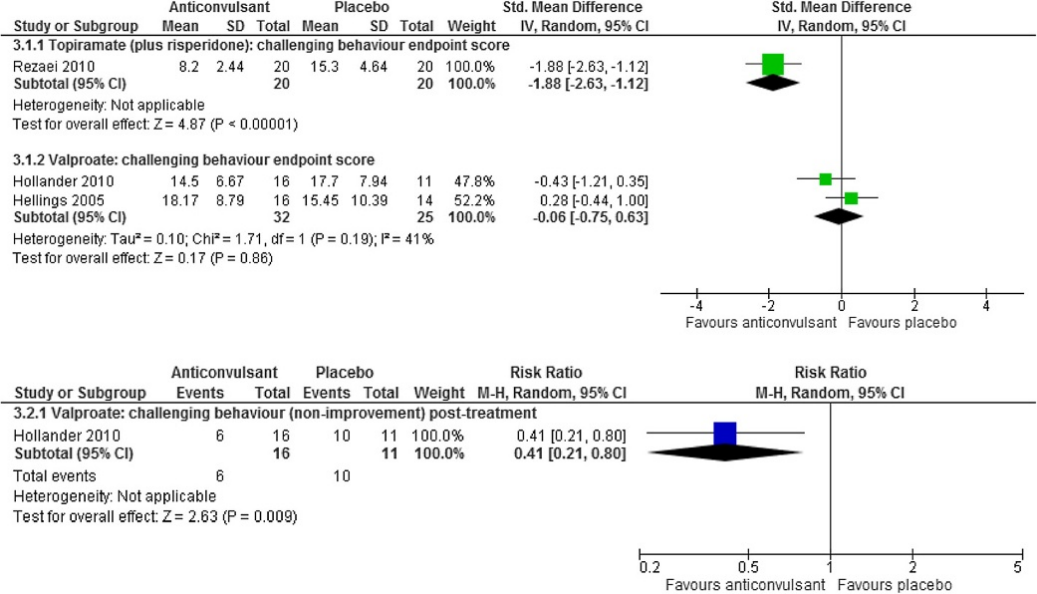
The effect of aripiprazole and risperidone on sedation in children with intellectual disabilities and challenging behaviour

There were greater levels of sedation among children treated with risperidone [37, 41, 45, 48, 49] and aripiprazole [43, 44] than those who received placebo (RR = 0.53, CI 0.42 to 0.68, *p* < 0.001 for risperidone; RR = 0.83, CI 0.76 to 0.91, *p* < 0.001 for aripiprazole; see Fig. 5). However, there was significant unexplained heterogeneity for the effect with risperidone (χ^2^ = 10.80, *p* = 0.03, I^2^ = 63 %).

One head-to-head study comparing risperidone to aripiprazole [46] found no difference in levels of sedation between groups (RR = 0.95, CI 0.74 to 1.22, *p* = 0.69).

A study comparing olanzapine to haloperidol [42] suggested that olanzapine increased drowsiness to a greater extent than haloperidol, but statistical estimates of this effect were inconclusive due to wide confidence intervals (RR = 0.25, CI 0.04 to 1.63, *p* = 0.15).

##### Prolactin

Risperidone was found to significantly increase prolactin. Studies showed that prolactin levels were over three times higher among children given risperidone than those given placebo [37, 45, 49] (SMD = 3.22, CI 1.68 to 4.75, *p* < 0.001; see Fig. 4). All studies demonstrated higher levels of prolactin among children treated with risperidone, compared to placebo, however this effect showed significant heterogeneity (χ^2^ = 30.66, *p* < 0.001, I^2^ = 93 %), likely due differences in the magnitude of effect across studies. More children had elevated prolactin levels with risperidone than placebo [37, 49] (RR 0.91, CI 0.85 to 0.97, *p* < 0.001). One child was found to have experienced oligomenorrhea - a prolactin-related adverse event – following treatment with risperidone [41], but analysis was inconclusive as to the relative risk of this event between groups (RR 0.97, CI 0.89 to 1.05, *p* = 0.44).

Evidence from two studies was inconclusive as to whether aripiprazole increased prolactin levels to a greater extent than placebo [43, 44] (RR 1.05, CI 0.99 to 1.10, *p* = 0.08).

##### Seizures

Evidence was inconclusive as to whether treatment with risperidone [45] or aripiprazole [43] increased the risk of seizures when compared to placebo (RR = 1.02, CI 0.97 to 1.08, *p* = 0.50 for risperidone; RR = 1.03, CI 0.98 to 1.08, *p* = 0.28 for aripiprazole).

A head-to-head study comparing risperidone to aripiprazole [46] found no difference in the rate of seizures between groups (RR = 1.03, CI 0.94 to 1.13, *p* = 0.49).

##### Study discontinuation

There were no significant differences in the rate of study discontinuation due to adverse events in placebo-controlled studies of risperidone [37, 41, 45, 48] or aripiprazole [43, 44] (RR = 0.99, CI 0.96 to 1.03, *p* = 0.76 for risperidone; RR = 0.96, CI 0.89 to 1.04, *p* = 0.33 for aripiprazole), or within a head-to-head study of risperidone compared to aripiprazole [46] (RR = 1.03, CI 0.94 to 1.13, *p* = 0.49).

#### Anticonvulsants

The efficacy of anticonvulsants for the treatment of challenging behaviour was explored in three studies, which included treatment with valproate and topiramate [39, 40, 47].

#### Efficacy outcomes

##### Challenging behaviour

**Fig. 6:**
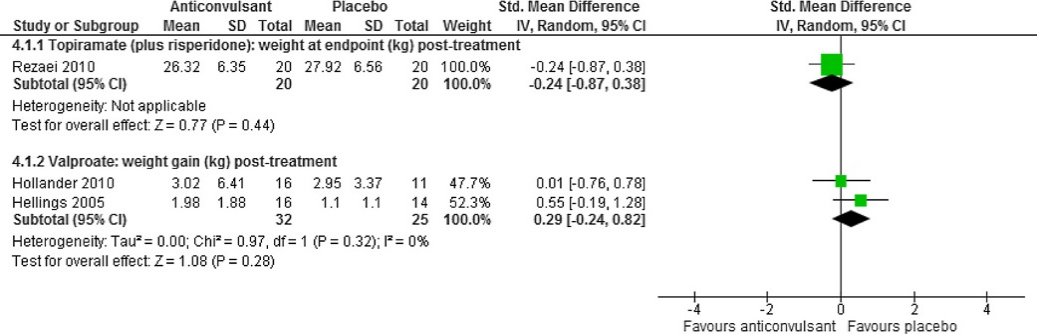
The effect of valproate and topiramate (added to risperidone) on challenging behaviour in children with intellectual disabilities

Two studies involving 57 children with autism investigated the effectiveness of valproate when compared with placebo and found mixed results [39, 40]. Evidence was inconclusive as to the effectiveness of valproate in reducing the severity of challenging behaviour based on ABC-I scores (SMD = −0.06, CI −0.75 to 0.63, *p* = 0.86) and there was significant unexplained heterogeneity in findings (χ^2^ = 1.71, *p* = 0.19, I^2^ = 41 %). However, a dichotomous measure of improvement incorporating CGI, ABC-I and Modified Overt Aggression Scale [53] scores, based on one of the studies [40] suggested that more children showed an improvement in challenging behaviour following treatment with valproate than those given placebo (RR = 0.41, CI 0.21 to 0.80, *p* = 0.009). Findings are presented in Fig. 6.

One study with 40 participants compared the effectiveness of treatment with topiramate combined with risperidone to a control condition in which participants received placebo with risperidone [47]. Risperidone was reportedly added to the intervention and control conditions in order for the study to obtain ethical approval. Combined treatment with topiramate and risperidone improved challenging behaviour to a greater extent than treatment with placebo plus risperidone (SMD = −1.88, CI −2.63 to −1.12, *p* < 0.001; see Fig. 6).

##### Other critical efficacy outcomes

No data were available for the effect of anticonvulsants on adaptive functioning, quality of life or service user and carer satisfaction.

#### Safety outcomes

There were no data for the effect of anticonvulsants on prolactin outcomes or seizures.

##### Weight

**Fig. 7:**
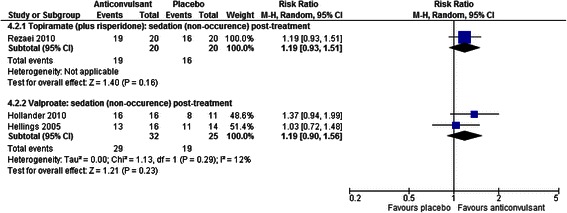
The effect of valproate and topiramate (added to risperidone) on weight in children with intellectual disabilities and challenging behaviour

Studies of valproate [39, 40] and of combined treatment with topiramate and risperidone [47] were inconclusive as to whether anticonvulsants increase weight gain, relative to placebo (SMD = 0.29, CI −0.24 to 0.82, *p* = 0.28 for weight change with valproate; SMD = −0.24, CI −0.87 to 0.38, p = 0.44 for weight at endpoint for topiramate and risperidone; see Fig. 7).

##### Sedation and somnolence

**Fig. 8:**
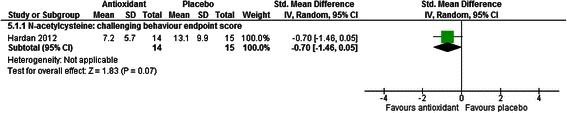
The effect of valproate and topiramate (added to risperidone) on sedation in children with intellectual disabilities and challenging behaviour

One placebo-controlled study of combined treatment with topiramate added to risperidone [47] and two exploring valproate [39, 40] were inconclusive as to the effect of these anticonvulsants on sedation (RR = 1.19, CI 0.93 to 1.51, *p* = 0.16 for topiramate with risperidone; RR = 1.19, CI 0.90 to 1.56, *p* = 0.23 for valproate; see Fig. 8).

##### Study discontinuation

Analysis of two studies was inconclusive as to whether treatment with valproate increased study discontinuation due to adverse events to a greater extent than placebo [39, 40] (RR = 0.95, CI 0.83 to 1.08, *p* = 0.41).

## Antioxidants

### Efficacy outcomes

#### Challenging behaviour

**Fig. 9:**
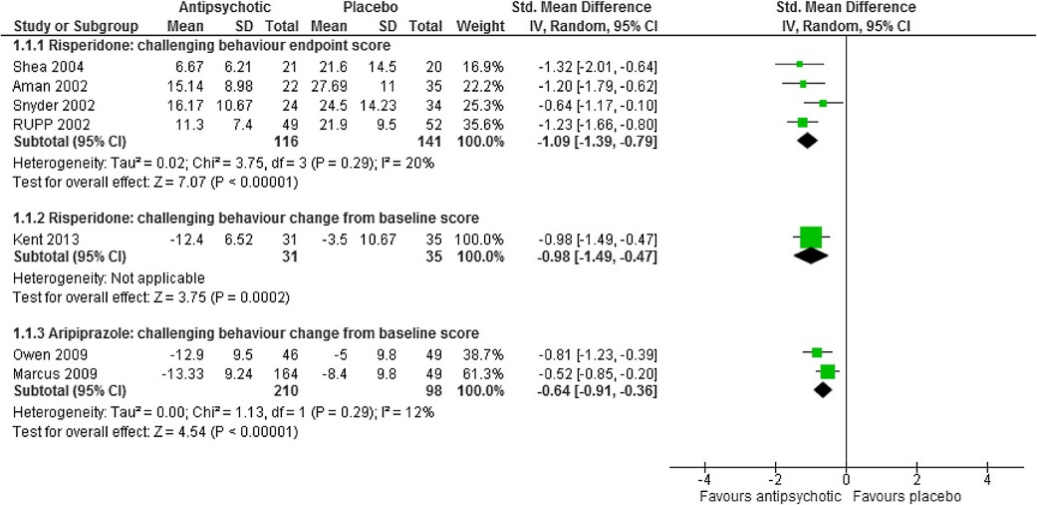
The effect of N-acetylcysteine on challenging behaviour in children with intellectual disabilities

N-acetylcysteine was compared with placebo for the treatment of challenging behaviour in a pilot study including 33 children with autism [38]. Children who received N-acetylcysteine showed lower levels of challenging behaviour than those given placebo, although confidence intervals crossed the line of no effect, suggesting that results were inconclusive (SMD = −0.70, CI −1.46 to 0.05, *p* = 0.07; see Fig. 9).

#### Other critical efficacy outcomes

No data were available for the effect of antioxidants on adaptive functioning, quality of life or service user and carer satisfaction.

### Safety outcomes

There were no data for the effect of antioxidants on weight, sedation, prolactin outcomes or seizures.

#### Study discontinuation

One small study was inconclusive as to the effect of N-acetylcysteine on study discontinuation due to adverse events [38] (RR = 0.93, CI 0.78 to 1.11, *p* = 0.42).

## Gamma-aminobutyric acid (GABA) analogues

### Efficacy outcomes

#### Challenging behaviour

One study involving 40 children with autism compared combined treatment with piracetam and risperidone to a control condition which included treatment with placebo plus risperidone [36]. It was not possible to include this study in meta-analysis, as data were not presented as means and standard deviations. However, the paper reports that challenging behaviour was significantly improved following treatment with piracetam plus risperidone when compared with the control condition (*F* = 5.85, d.f. = 1, *p* = 0.02).

#### Other critical efficacy outcomes

No data were available for the effect of GABA analogues on adaptive functioning, quality of life or service user and carer satisfaction.

### Safety outcomes

There were no data for the effect of GABA analogues on weight, prolactin, seizures or study discontinuation.

#### Sedation

Based on one study, analysis was inconclusive as to the effect of piracetam on daytime drowsiness [36] (RR = 1.18, CI 0.71 to 1.97, *p* = 0.52).

## Discussion

### Main findings

This systematic review suggests that antipsychotic medications may be effective in reducing challenging behaviour among children with intellectual disabilities in the short-term. Aripiprazole and risperidone reduced post-treatment challenging behaviour with effect sizes in the moderate to large range. However, there was a lack of evidence regarding the long-term effectiveness of antipsychotic medication for reducing challenging behaviour and the quality of evidence was low. Evidence for the effectiveness of anticonvulsants and antioxidants was inconclusive. GABA analogues were reportedly effective for the treatment of challenging behaviour in one small study.

This review also demonstrates that antipsychotic medication was associated with a range of side effects among children including significant weight gain, increased prolactin levels and sedation. Such side effects were apparent after six to twelve weeks of treatment. Available evidence did not permit exploration of the long term consequences of these side effects, or of any other effects that may emerge with prolonged treatment. However, existing evidence confirms that a variety of side effects have been noted following the use of antipsychotic medication including somnolence, extrapyramidal symptoms, increased prolactin concentrations, significant weight gain and cardiovascular dysfunction [37, 43, 49, 54]. Elevated prolactin levels may be of particular concern for children, as hyperprolactinemia can adversely affect long-term physical and sexual development, having been associated with conditions such as amenorrhea, erectile dysfunction and osteoporosis [55, 56]. Despite evidence of significant side effects, antipsychotic medications are still commonly prescribed for the treatment of behavioural disturbance among children and adolescents with intellectual disability [57].

Notably, many of the drugs investigated in this review have been used outside of their licenced indication. This accords with evidence that suggests that off-label prescribing may be as high as 46 % among individuals with intellectual disabilities [13]. Only aripiprazole and risperidone have been approved by the U.S Food and Drug Administration for irritability associated with autistic disorder in paediatric patients [58, 59]. A recent audit suggested that nearly three quarters of individuals with intellectual disabilities were prescribed antipsychotic medication and a third of these were prescribed to treat challenging behaviour [12].

Given the possibility that individuals with intellectual disabilities may be more susceptible to drug side-effects, and the lack of firm evidence for the effectiveness of anticonvulsants, GABA analogues and antioxidants, clinicians must be cautious when choosing to prescribe drugs off-label for the treatment of challenging behaviour [60, 61].

### Strengths and limitations of the review

The strengths of this review and meta-analysis include a rigorous methodology, and investigation of a wide range of pharmacological interventions among children with intellectual disabilities. A review of this type and scale has not previously been conducted in this area. Furthermore, the review incorporated previously unpublished data, further adding to the utility of this analysis.

Nevertheless, the results of this review must be interpreted with caution as most outcomes were based on a small number of studies, the quality of evidence ranged from low to very low and information was only available for short-term outcomes. Study quality was compromised most commonly by small sample sizes, but also by inadequate reporting of methods of blinding, randomisation and allocation concealment by study authors. Therefore, conclusions drawn from this review are susceptible to change as further evidence becomes available. Secondly, the majority of participants in the review were diagnosed with autism. While autism is frequently associated with a concurrent diagnosis of intellectual disabilities, and the review included disaggregated data sets of individuals with intelligence test score of less than 70, where available, it remains possible that some participants did not meet criteria for intellectual disability. It is not clear how this would affect the interpretation of findings.

## Conclusions

Available evidence suggests that antipsychotic medications may be effective for reducing challenging behaviour in the short-term among children with intellectual disabilities. Findings from this review must be interpreted with caution as studies were generally of low quality and most outcomes were based on a small number of studies. Further multicentre research utilising randomised designs and health related quality of life is needed to determine the clinical and cost effectiveness of psychotropic medication for challenging behaviour either alone or in addition to psychosocial interventions. Longitudinal follow up of participants can also shed further light on issues of safety especially as such treatments may be taken over extended periods of time.

## Additional files

Additional file 1: **PRISMA checklist.** (PDF 251 kb)
Additional file 2: **Medline search string.** (PDF 314 kb)
Additional file 3: **GRADE evidence profiles.** (PDF 199 kb)

## Abbreviations

ABC-I: Aberrant Behaviour Checklist: irritability subscale
ABC total: Aberrant Behaviour Checklist total score
ASD: Autism Spectrum Disorders
BEI: British Education Index
BH: Bronwyn Harrison
CB: challenging behaviour
CDSR: Cochrane Database of Systematic Reviews
CI: confidence interval
CENTRAL: Cochrane Central Register of Controlled Trials
CGI-I: Clinical Global Impressions: Improvement
CM: Cheryl McQuire
CPRS-H: Children’s Psychiatric Rating Scale: hyperactivity subscale
DARE: Cochrane Database of Abstracts of Reviews of Effects
ERIC: Education Resources Information Centre
GABA: gamma-aminobutyric acid
GRADE: Grading of Recommendations Assessment, Development and Evaluation
IBSS: International Bibliography of the Social Sciences
IDD: intellectual developmental disorder
NCBRF: Nisonger Child Behaviour Rating Form: conduct problems subscale
NICE: National Institute for Health and Care Excellence
RCT: randomised controlled trial
RR: risk ratio
RUPP: Research Units on Pediatric Psychopharmacology
SMD: standardised mean difference
SP: Stephen Pilling
SSCI: Social Sciences Citation Index
χ2: Chi-squared
