# Erratum to: Pharmacological interventions for challenging behaviour in children with intellectual disabilities: a systematic review and meta-analysis

**DOI:** 10.1186/s12888-015-0704-6

**Published:** 2016-01-07

**Authors:** Cheryl McQuire, Angela Hassiotis, Bronwyn Harrison, Stephen Pilling

**Affiliations:** National Collaborating Centre for Mental Health, Royal College of Psychiatrists, 21 Prescot Street, London, E1 8BB UK; Division of Psychiatry, University College London, Charles Bell House, 1st and 2nd Floor, 67-73 Riding House Street, London, W1W 7EJ UK; Centre for Outcomes Research and Effectiveness, University College London, 1-19 Torrington Place, London, WC1E 7HB UK

After the publication of the original article [1] in BMC Psychiatry it was brought to our attention that Figs. 3, 4, 5, 6, 7, 8 and 9 were ordered incorrectly. Please find the correct order of the figures and citations below.

**Fig. 3:**
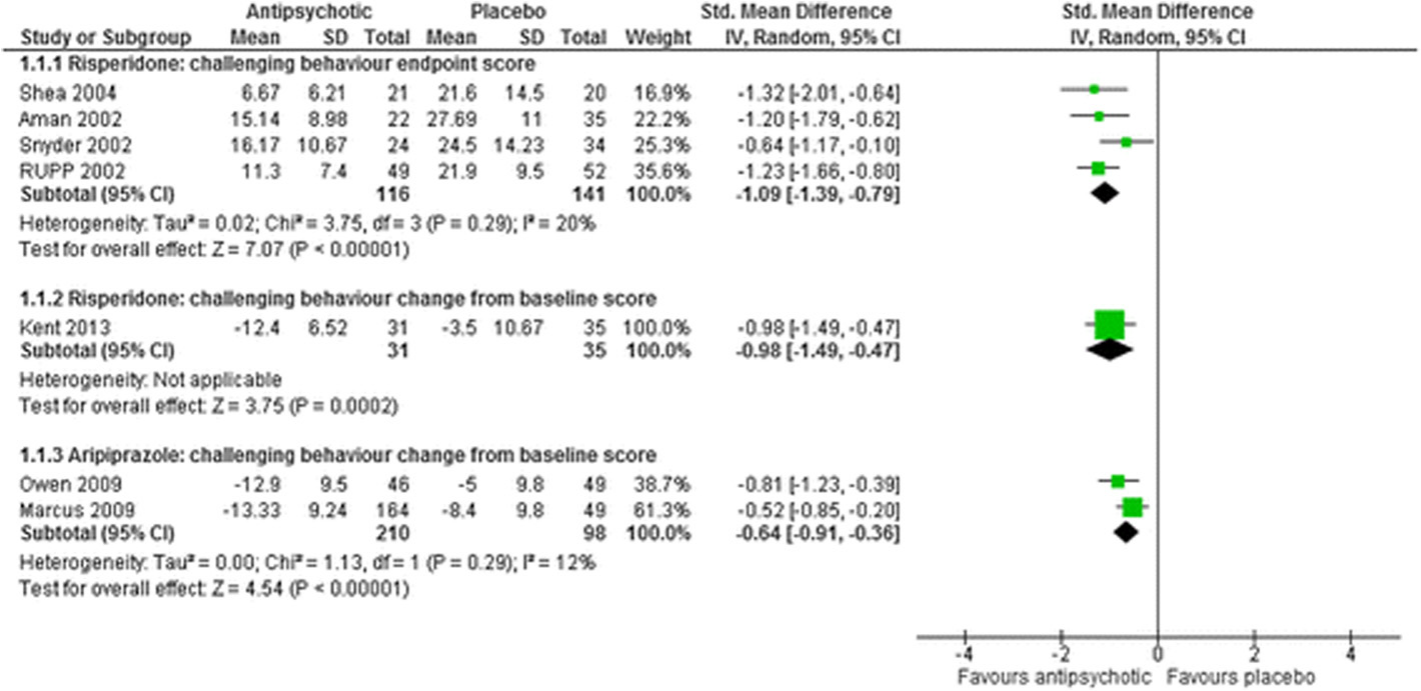
The effect of risperidone and aripiprazole on challenging behaviour in children with intellectual disabilities

**Fig. 4:**
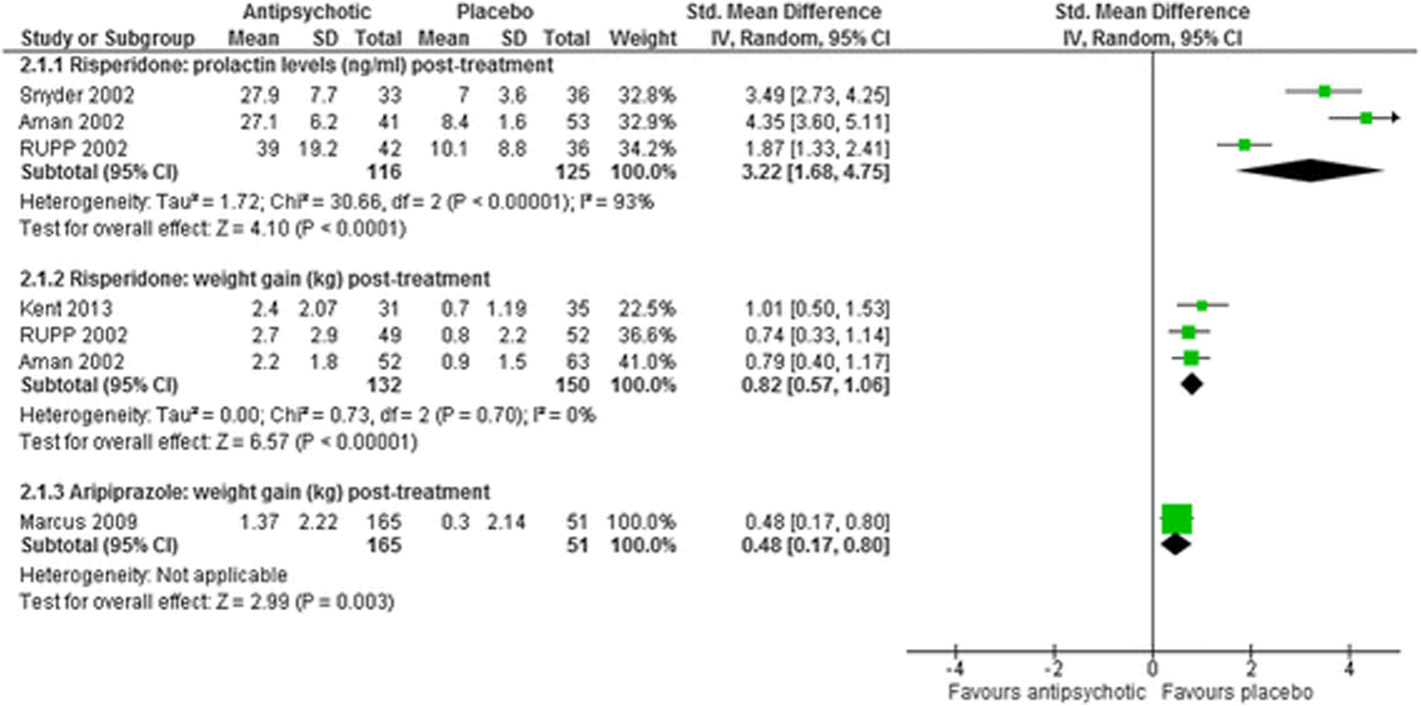
The effect of aripiprazole and risperidone on weight and prolactin levels in children with intellectual disabilities and challenging behaviour

**Fig. 5:**
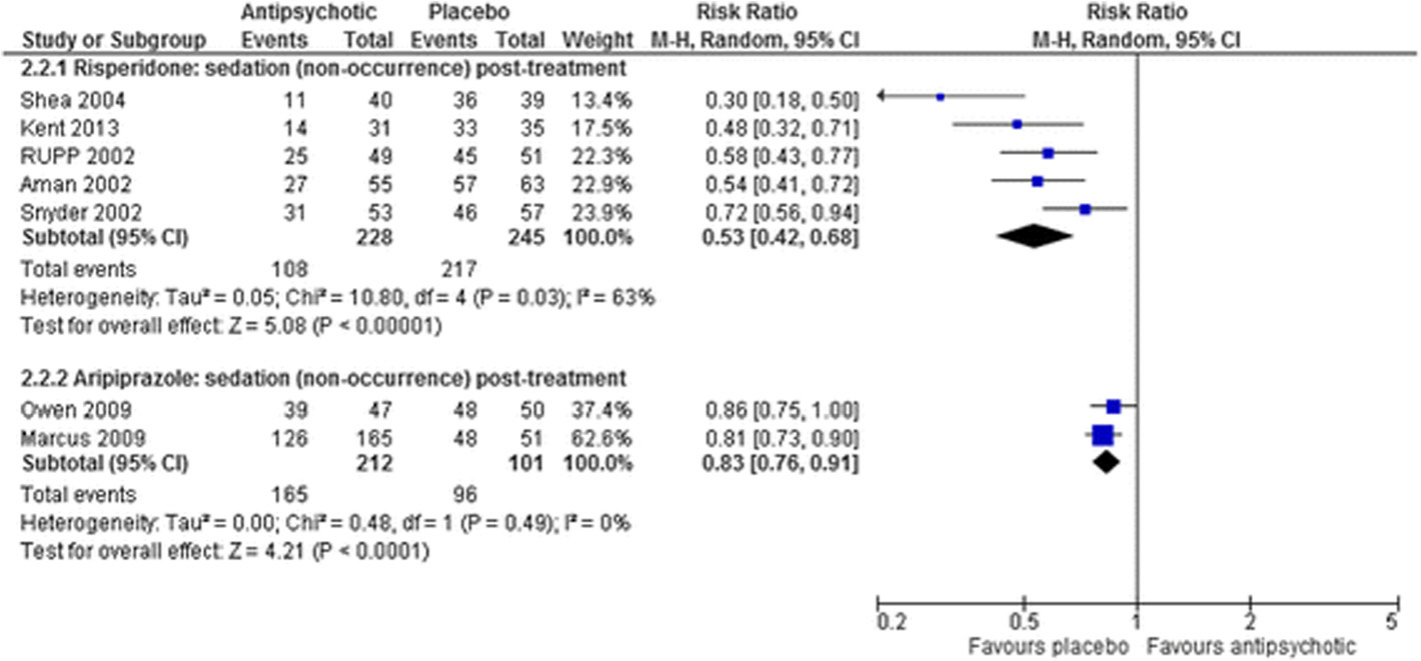
The effect of aripiprazole and risperidone on sedation in children with intellectual disabilities and challenging behaviour

**Fig. 6:**
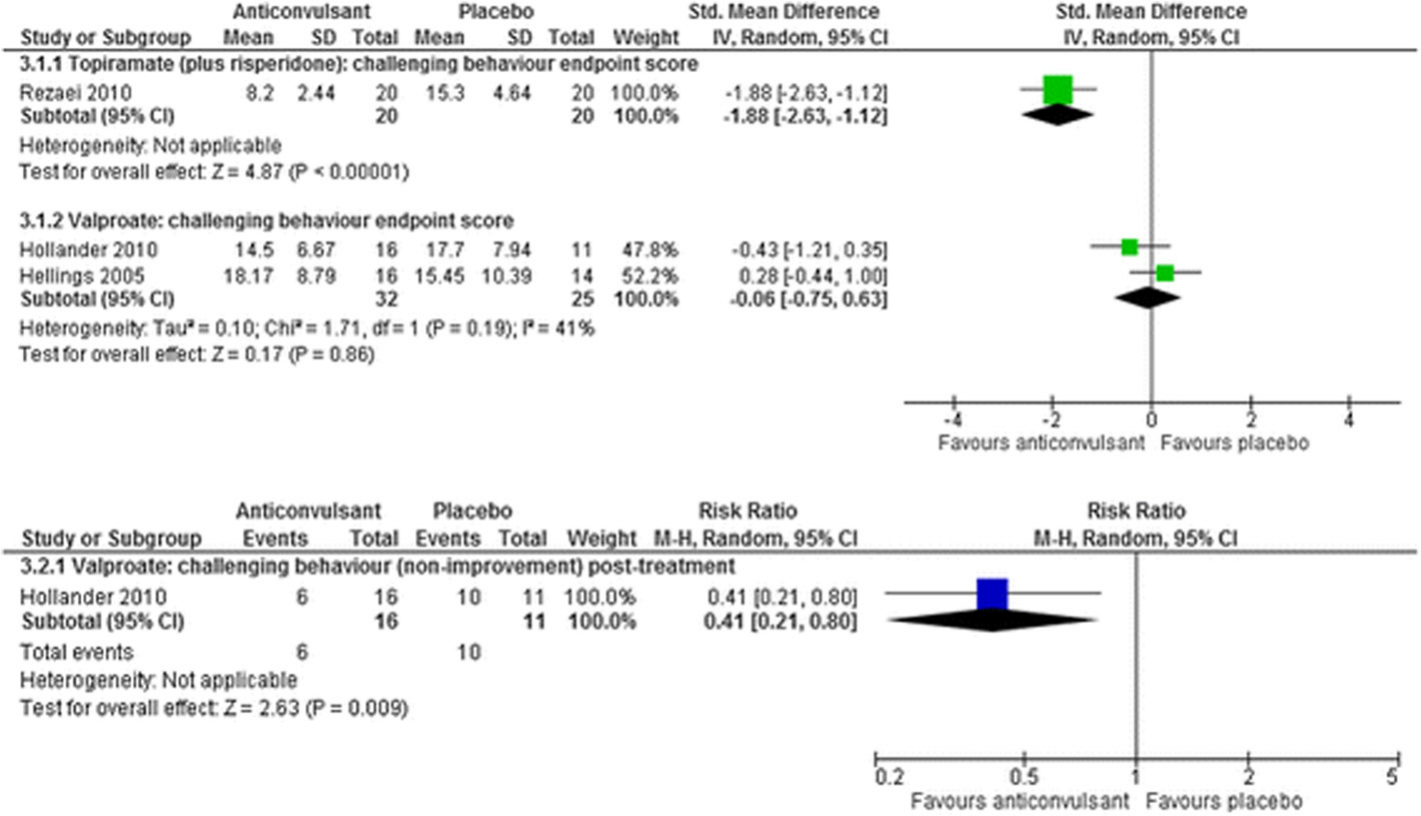
The effect of valproate and topiramate (added to risperidone) on challenging behaviour in children with intellectual disabilities

**Fig. 7:**
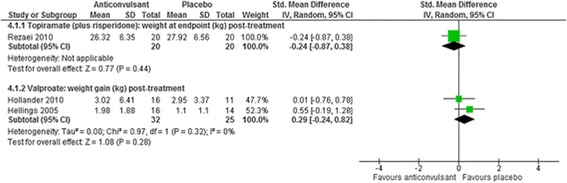
The effect of valproate and topiramate (added to risperidone) on weight in children with intellectual disabilities and challenging behaviour

**Fig. 8:**
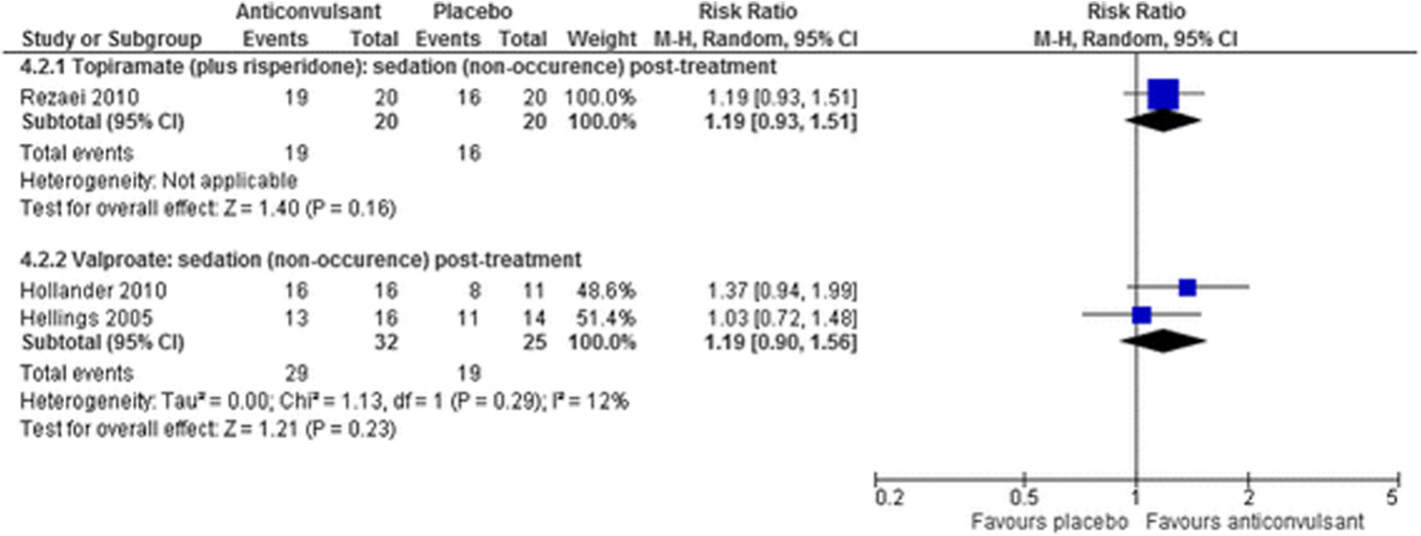
The effect of valproate and topiramate (added to risperidone) on sedation in children with intellectual disabilities and challenging behaviour

**Fig. 9:**
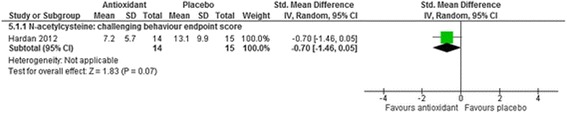
The effect of N-acetylcysteine on challenging behaviour in children with intellectual disabilities
