# Supplementary material for: Pharmacological interventions for challenging behaviour in children with intellectual disabilities: a systematic review and meta-analysis
Source: BMC Psychiatry. 2015 Nov 26;15:303. doi: 10.1186/s12888-015-0688-2 (PMC4662033; doi:10.1186/s12888-015-0688-2)
Supplement: Additional file 2: — Medline search string. (PDF 314 kb) [file 12888_2015_688_MOESM2_ESM.pdf]

Pharmacological [generic – this answered all RQs answerable via RCTs).

Medline – OVID SP

1	developmental disabilities/ or exp intellectual disability/ or exp learning disorders/ or mentally disabled persons/
2	((intellect* adj (deficien* or difficult* or disab* or disorder* or impair* or handicap* or incapacit* or sub?average or sub?norm*)) or (low*2 adj2 intellect*) or (learning adj (deficien* or difficult* or disab* or disorder* or handicap* or impair* or incapacit* or handicap* or sub?average or sub?norm*)) or (mental* adj (deficien* or disab* or handicap* or impair* or handicap* or incapacit* or retard* or sub?average or sub?norm*))).ti,ab.
3	((sub?average or sub?normal*) adj3 (child* or cognit* or intel*)).tw.
4	((development* or neurodevelopment*) adj disab*).tw.
5	(education* adj5 sub?norm*).tw.
6	(cretin* or feeble?minded* or imbecil* or moron*).tw.
7	(handicap* or multiple disabilit* or developmental delay*).ti,ab.
8	angelman syndrome/ or down syndrome/ or fragile x syndrome/
9	(((angelman or happy puppet) adj2 syndrom*) or (down*1 adj (disease or syndrome*)) or bell martin or fragile x or gillian turner or martin bell or turner gillian or x chromosome fragility or (escalante* adj (disease or syndrome*))).ti,ab.
10	autistic disorder/ or asperger syndrome/
11	(autis* or asperger* or pervasive developmen* disorder* or rett*1).ti,ab.
12	((communicat* adj2 impair*) or ((complex or high support) adj3 needs) or dyslex* or ((language or speech) adj2 (delay* or disorder*))).ti,ab. or dyslexi*.sh.
13	or/1-12
14	behav*.ti,ab,hw.
15	acting out/ or exp domestic violence/ or exp aggression/ or exp anger/ or dangerous behavior/ or hate/ or hostility/ or sexual harrassment/ or torture/ or violence/
16	(abus* or aggress* or agitat* or agonistic or anger or angry or assault* or attack* or bizarre or combative or danger* or destruct* or disrupt* or disturb* or harass* or hostil* or intimidat* or rage* or threat* or un?toward or violen*).ti,ab.
17	overdose/ or self-injurious behavior/ or self mutilation/ or stereotypic movement disorder/ or suicide/ or suicidal ideation/ or suicide, attempted/
18	(auto?aggress* or auto?mutilat* or cutt* or overdos* or para?suicid* or (self adj2 cut*) or self?damag* or self?destruct* or self?harm* or self?hurt* or self?inflict* or self?injur* or self?mutilat* or self?poison* or self?wound* or suicid*).ti,ab.
19	impulse control disorders/
20	((impulse control disorder*) or (explosive adj2 disorder)).ti,ab.
21	exp child behavior/ or conduct disorder/
22	(conduct* or (oppositional adj3 (defiant* or disorder*))).ti,ab.
23	(attenti* or disrupt* or impulsiv* or inattenti*).sh.

24	(attenti* adj3 (adolescen* or adult* or behav* or child* or class or classes or classroom* or condition* or difficult* or disorder* or learn* or people or person* or poor or problem* or process* or youngster*)).ti,ab.
25	(impulsiv* or inattentiv* or adhd or (attenti* adj3 deficit*) or hyperactiv* or (hyper adj1 activ*) or hyperkin*).ti,ab. or (overactiv*.tw. not overactive bladder*.ti.)
26	"conflict (psychology)"/ or firesetting behavior/ or fraud/ or juvenile delinquency/ or rape/ or sex offences/ or theft/ or torture/
27	((adjust* adj2 (difficult* or problem*)) or anti?social* or arson or asbo or asocial or bully* or bullie* or callous* or delinquen* or deviant* or (fire* adj2 (alight or set* or start*)) or mal?adjust* or psychopath* or pyromani* or shop?lift* or steal* or temper*1 or theft* or unemotional trait* or vandali* or (sex* adj2 (abus* or harass* or offen*)) or ((social or unemotional) adj (difficult* or problem*)) or non?complian*).ti,ab.
28	(repetitiv* or self stimulat* or stereotyp* or sucking).hw.
29	(((crumpl* or tear*) adj2 paper*) or fingering* or (finger* adj2 (flex* or tap*)) or flapping or knee shaking or humming or ((loud* or nois*) adj2 (instrument* or vocali*)) or lip pucker* or repetitive* or ritualistic or (rocking or (body adj2 rock*)) or (self stimulat* or selfstimulat*) or staring or stemming or stereotyp* or (thumb adj2 suck*) or (wav* adj2 (arm* or hand*))).ti,ab.
30	wander*.ti,ab,hw.
31	withdrawn*.ti,ab,hw.
32	or/14-31
33	13 and 32

<u>Systematic review study design filter: Medline – OVID SP</u>

1	meta analysis.sh,pt. or "meta-analysis as topic"/ or "review literature as topic"/
2	(exp databases, bibliographic/ or (((electronic or computer$ or online) adj database$) or bids or cochrane or embase or index medicus or isi citation or medline or psyclit or psychlit or scisearch or science citation or (web adj2 science)).ti,ab.) and (review$.ti,ab,sh,pt. or systematic$.ti,ab.)
3	((analy$ or assessment$ or evidence$ or methodol$ or quantitativ$ or systematic$) adj2 (overview$ or review$)).tw. or ((analy$ or assessment$ or evidence$ or methodol$ or quantitativ$ or systematic$).ti. and review$.ti,pt.) or (systematic$ adj2 search$).ti,ab.
4	(metaanal$ or meta anal$).ti,ab.
5	(research adj (review$ or integration)).ti,ab.
6	reference list$.ab.
7	bibliograph$.ab.
8	published studies.ab.
9	relevant journals.ab.
10	selection criteria.ab.
11	(data adj (extraction or synthesis)).ab.
12	(handsearch$ or ((hand or manual) adj search$)).ti,ab.
13	(mantel haenszel or peto or dersimonian or der simonian).ti,ab.
14	(fixed effect$ or random effect$).ti,ab.
15	((pool$ or combined or combining) adj2 (data or trials or studies or results)).ti,ab.
16	or/1-15

1	exp clinical trial/ or exp “clinical trials as topic”/ or cross-over studies/ or double-blind method/ or placebos/ or random allocation/ or single-blind method/
2	(clinical adj2 trial$).ti,ab.
3	(crossover or cross over).ti,ab.
4	(((single$ or doubl$ or trebl$ or tripl$) adj2 blind$) or mask$ or dummy or doubleblind$ or singleblind$ or trebleblind$ or tripleblind$).ti,ab.
5	(placebo$ or random$).ti,ab.
6	animal$/ not human$/ use mesz, prem
7	or/1-5 not 6
