# Supplementary material for: Pharmacological interventions for challenging behaviour in children with intellectual disabilities: a systematic review and meta-analysis
Source: BMC Psychiatry. 2015 Nov 26;15:303. doi: 10.1186/s12888-015-0688-2 (PMC4662033; doi:10.1186/s12888-015-0688-2)
Supplement: Additional file 3: — GRADE evidence profiles. (PDF 199 kb) [file 12888_2015_688_MOESM3_ESM.pdf]

1 Clinical Evidence Profiles (GRADE)

1.1 Pharmacological interventions aimed at reducing and managing behaviour that challenges

1.1.1 Risperidone versus placebo in children and young people

Table 1: Risperidone versus placebo in children and young people

Quality assessment							Summary of Findings				
Participants (studies) Follow up	Risk of bias	Inconsistency	Indirectness	Imprecision	Publication bias	Overall quality of evidence	Study event rates (%)		Relative effect (95% CI)	Anticipated absolute effects	
							With placebo	With risperidone		Risk with placebo	Risk difference with risperidone (95% CI)
**Targeted behaviour that challenges (severity) - post-treatment** (measured with: End-point score; Better indicated by lower values)											
257 (4 studies)	serious[1]	no serious inconsistency	no serious indirectness	serious[2]	undetected	⊕⊕⊖⊖ **LOW**[1,2] due to risk of bias, imprecision	141	116	-		The mean targeted behaviour that challenges (severity) - post-treatment in the intervention groups was **1.09 standard deviations lower** (1.39 to 0.79 lower)
**Targeted behaviour that challenges (severity) - post-treatment** (measured with: Change score; Better indicated by lower values)											
66 (1 study)	serious[3]	no serious inconsistency	serious[4]	very serious[5]	undetected	⊕⊖⊖⊖ **VERY LOW**[3,4,5] due to risk of bias, indirectness, imprecision	35	31	-		The mean targeted behaviour that challenges (severity) - post-treatment in the intervention groups was **0.98 standard deviations lower** (1.49 to 0.47 lower)
**Targeted behaviour that challenges (severity, non-improvement) - post-treatment**											

153<br>(2 studies)	serious[1]	no serious inconsistency	no serious indirectness	serious[2]	undetected	⊕⊕⊝⊝<br>**LOW**[1,2]<br>due to risk of bias, imprecision	68/80<br>(85%)	25/73<br>(34.2%)	**RR 0.42**<br>(0.28 to 0.64)	**850 per 1000**	**493 fewer per 1000**<br>(from 306 fewer to 612 fewer)
**Adaptive functioning (social) - post-treatment** (measured with: Nisonger Child Behaviour Rating Form - Social Compliance[6]; Better indicated by higher values)											
155<br>(3 studies)	serious[1]	no serious inconsistency	no serious indirectness	serious[2]	undetected	⊕⊕⊝⊝<br>**LOW**[1,2]<br>due to risk of bias, imprecision	88	67	-		The mean adaptive functioning (social) - post-treatment in the intervention groups was<br>**0.86 standard deviations higher**<br>(0.42 to 1.3 higher)
[1] Most information is from studies at moderate risk of bias<br>[2] Optimal information size not met<br>[3] Crucial limitation for one criterion or some limitations for multiple criteria sufficient to lower ones confidence in the estimate of effect<br>[4] Applicability - different populations<br>[5] Optimal information size not met; small, single study<br>[6] Combined adaptive social and compliant/calm subscales											

1.1.2 Aripiprazole versus placebo in children and young people

Table 2: Aripiprazole versus placebo in children and young people

Quality assessment							Summary of Findings				
**Participants (studies) Follow up**	**Risk of bias**	**Inconsistency**	**Indirectness**	**Imprecision**	**Publication bias**	**Overall quality of evidence**	**Study event rates (%)**		**Relative effect** (95% CI)	**Anticipated absolute effects**	
							**With placebo**	**With aripiprazole**		**Risk with placebo**	**Risk difference with aripiprazole** (95% CI)
**Targeted behaviour that challenges (severity) - post-treatment** (Better indicated by lower values)											
308<br>(2 studies)	serious[1]	no serious inconsistency	serious[2]	serious[3]	undetected	⊕⊝⊝⊝<br>**VERY LOW**[1,2,3]	98	210	-		The mean targeted behaviour that challenges (severity) - post-treatment in the intervention groups was

						due to risk of bias, indirectness, imprecision					**0.64 standard deviations lower** (0.91 to 0.36 lower)
**Targeted behaviour that challenges (severity, non-improvement) - post-treatment**											
308 (2 studies)	serious[1]	no serious inconsistency	serious[2]	serious[3]	undetected	⊕⊖⊖⊖ **VERY LOW**[1,2,3] due to risk of bias, indirectness, imprecision	74/98 (75.5%)	100/210 (47.6%)	**RR 0.65** (0.5 to 0.84)	**755 per 1000**	**264 fewer per 1000** (from 121 fewer to 378 fewer)
**Quality of life - post-treatment** (Better indicated by higher values)											
243 (2 studies)	serious[1]	very serious[4]	serious[2]	serious[3]	undetected	⊕⊖⊖⊖ **VERY LOW**[1,2,3,4] due to risk of bias, inconsistency, indirectness, imprecision	76	167	**-**		The mean quality of life - post-treatment in the intervention groups was **0.6 standard deviations higher** (0.17 lower to 1.37 higher)

[1] Most information is from studies at moderate risk of bias
[2] Applicability - different populations
[3] Optimal information size not met
[4] I2 > 75%

1.1.3 Aripiprazole versus risperidone in children and young people

Table 3: Aripiprazole versus risperidone in children and young people

Quality assessment							Summary of Findings				
**Participants (studies) Follow up**	**Risk of bias**	**Inconsistency**	**Indirectness**	**Imprecision**	**Publication bias**	**Overall quality of evidence**	**Study event rates (%)**		**Relative effect** (95% CI)	**Anticipated absolute effects**	
							**With risperidone**	**With aripiprazole**		**Risk with risperidone**	**Risk difference with aripiprazole** (95% CI)
**Targeted behaviour that challenges (severity) - post-treatment** (Better indicated by lower values)											

59<br>(1 study)	serious[1]	no serious inconsistency	serious[2]	very serious[3]	undetected	⊕⊖⊖⊖<br>**VERY LOW**[1,2,3]<br>due to risk of bias, indirectness, imprecision	30	29	-		The mean targeted behaviour that challenges (severity) - post-treatment in the intervention groups was<br>**0.38 standard deviations higher**<br>(0.14 lower to 0.9 higher)
[1] Crucial limitation for one criterion or some limitations for multiple criteria sufficient to lower ones confidence in the estimate of effect<br>[2] Applicability - different populations<br>[3] Optimal information size not met; small, single study											

1.1.4 Olanzapine versus haloperidol in children and young people

Table 4: Olanzapine versus haloperidol in children and young people

Quality assessment							Summary of Findings				
Participants (studies) Follow up	Risk of bias	Inconsistency	Indirectness	Imprecision	Publication bias	Overall quality of evidence	Study event rates (%)		Relative effect (95% CI)	Anticipated absolute effects	
							With haloperidol	With oanzapine		Risk with haloperidol	Risk difference with olanzapine (95% CI)
**Targeted behaviour that challenges (severity) - post-treatment** (Better indicated by lower values)											
12<br>(1 study)	very serious[1]	no serious inconsistency	no serious indirectness	very serious[2]	undetected	⊕⊖⊖⊖<br>**VERY LOW**[1,2]<br>due to risk of bias, imprecision	6	6	-		The mean targeted behaviour that challenges (severity) - post-treatment in the intervention groups was<br>**1.4 standard deviations lower**<br>(2.73 to 0.08 lower)
[1] Crucial limitation for one or more criteria sufficient to substantially lower ones confidence in the estimate of effect.<br>[2] Optimal information size not met; small, single study											

1.1.5 Topiramate (plus risperidone) versus placebo (plus risperidone) in children and young people

Table 5: Topiramate (plus risperidone) versus placebo (plus risperidone) in children and young people

Quality assessment							Summary of Findings				
Participants (studies) Follow up	Risk of bias	Inconsistency	Indirectness	Imprecision	Publication bias	Overall quality of evidence	Study event rates (%)		Relative effect (95% CI)	Anticipated absolute effects	
							With placebo plus risperidone	With topiramate plus risperidone		Risk with placebo plus risperidone	Risk difference with topiramate plus risperidone (95% CI)
**Targeted behaviour that challenges (severity) - post-treatment** (Better indicated by lower values)											
40 (1 study)	no serious risk of bias	no serious inconsistency	serious[1]	very serious[2]	undetected	⊕⊖⊖⊖ **VERY LOW**[1,2] due to indirectness, imprecision	20	20	-		The mean targeted behaviour that challenges (severity) - post-treatment in the intervention groups was **1.88 standard deviations lower** (2.63 to 1.12 lower)

[1] Applicability - different populations
[2] Optimal information size not met; small, single study

1.1.6 Valproate versus placebo in children and young people

Table 6: Topiramate (plus risperidone) versus placebo (plus risperidone) in children and young people

Quality assessment							Summary of Findings				
Participants (studies) Follow up	Risk of bias	Inconsistency	Indirectness	Imprecision	Publication bias	Overall quality of evidence	Study event rates (%)		Relative effect (95% CI)	Anticipated absolute effects	
							With placebo	With valproate		Risk with placebo	Risk difference with valproate (95% CI)

**Targeted behaviour that challenges (severity) - post-treatment** (Better indicated by lower values)											
57<br>(2 studies)	serious[1]	serious[2]	no serious indirectness	serious[3]	undetected	⊕⊖⊖⊖<br>**VERY LOW**[1,2,3]<br>due to risk of bias, inconsistency, imprecision	25	32	**-**		The mean targeted behaviour that challenges (severity) - post-treatment in the intervention groups was<br>**0.06 standard deviations lower**<br>(0.75 lower to 0.63 higher)
**Targeted behaviour that challenges (severity, non-improvement) - post-treatment**											
27<br>(1 study)	serious[4]	no serious inconsistency	no serious indirectness	very serious[5]	undetected	⊕⊖⊖⊖<br>**VERY LOW**[4,5]<br>due to risk of bias, imprecision	10/11<br>(90.9%)	6/16<br>(37.5%)	**RR 0.41**<br>(0.21 to 0.8)	**909 per 1000**	**536 fewer per 1000**<br>(from 182 fewer to 718 fewer)

[1] Most information is from studies at moderate risk of bias
[2] I2 > 40%
[3] Optimal information size not met
[4] Crucial limitation for one criterion or some limitations for multiple criteria sufficient to lower ones confidence in the estimate of effect
[5] Optimal information size not met; small, single study

1.1.7 N-acetylcysteine versus placebo in children and young people

Table 7: N-acetylcysteine versus placebo in children and young people

Quality assessment							Summary of Findings				
Participants (studies) Follow up	Risk of bias	Inconsistency	Indirectness	Imprecision	Publication bias	Overall quality of evidence	Study event rates (%)		Relative effect (95% CI)	Anticipated absolute effects	
							With placebo	With N-acetylcysteine (NAC)		Risk with placebo	Risk difference with N-acetylcysteine (NAC) (95% CI)
**Targeted behaviour that challenges (severity) - post-treatment** (Better indicated by lower values)											
29<br>(1 study)	serious[1]	no serious inconsistency	serious[2]	very serious[3]	undetected	⊕⊖⊖⊖<br>**VERY LOW**[1,2,3]	15	14	-		The mean targeted behaviour that challenges (severity) - post-treatment in the intervention groups was

						due to risk of bias, indirectness, imprecision				**0.70 standard deviations lower** (1.46 lower to 0.05 higher)

[1] Crucial limitation for one criterion or some limitations for multiple criteria sufficient to lower ones confidence in the estimate of effect
[2] Applicability - different populations
[3] Optimal information size not met; small, single study
